# Self-control of states: bridging social psychology to international relations discourses

**DOI:** 10.3389/fsoc.2024.1426476

**Published:** 2024-09-25

**Authors:** Bama Andika Putra

**Affiliations:** ^1^School of Sociology, Politics, and International Studies, University of Bristol, Bristol, United Kingdom; ^2^Department of International Relations, Universitas Hasanuddin, Makassar, Indonesia

**Keywords:** self-control theory, sociology, social psychology, states, international relations

## Abstract

Why do states respond non-coercively in the face of crisis? Existing scholarship within international relations has stagnated in its conclusions regarding understanding this occurrence. This perspective article attempts to bridge the self-control theory of social psychology to provide a more nuanced understanding of why states self-refrain themselves from taking aggressive retaliatory foreign policies in state-to-state crises. It argues the importance of cognitive-affective units, such as encodings, expectancies, beliefs, goals, values, and self-regulatory plans, as the sociological interpretation of why states are committed to pursuing delayed rewards. It builds upon existing sociological theories adopted in international relations scholarship, such as state identities and role conceptions, and further considers the social psychology variables detrimental in self-control theories, and argues for its relevance to decompose the ability of a state to prioritize delayed gratification over immediate awards in tensions faced.

## 1 Introduction

An empirical puzzle left unexplored in the study of international relations is why states decide to refrain from aggressive foreign policies amid a crisis. Existing scholarship within foreign policy studies has referenced the importance of looking into the national interests of state actors and how they connect to present-time decisions (Kaarbo, 2015; Baylis et al., 2019). However, the decision to self-control actions for future gains has somewhat been excluded from academic inquiries.

Indeed, this empirical puzzle has been recurring in many of the world’s hotspots. In the South China Sea crisis involving China and the secondary states of Southeast Asia, claimant states have been careful not to elevate tensions by the adoption of peaceful yet decisive maneuvers at sea to maintain sovereign claims over the contested maritime borders (Fravel, 2011; Yahuda, 2013; Qi, 2019; Putra, 2023a). In a recent crisis involving Israel and Iran, responses have been instead limited compared to the invocation of the self-defense measures that states have shown during the Cold War and in the early 21st century (Landale, 2024; McCarthy and John, 2024; Millar, 2024). What is it that state actors consider when they decide to self-control themselves from aggressive actions in international relations?

Two dominant schools of thought have attempted to make sense of a state’s responses to the crisis. Under structuralism, the variables usually considered are a state’s national interests and a pessimistic account of how states behave in the international system (Taliaferro, 2000; Hoffman, 2007; Guzzini, 2013). Thus, when states refrain from aggressive retaliatory actions, it is usually because what is at stake holds less importance for the state. Meanwhile, constructivism interprets such actions by raising or de-escalating the significance of particular issues within its domestic constituencies and focusing on the social construction of the problems associated with the crisis (Hopf, 1998; Guzzini and Leander, 2006). Thus, the decision to cooperate in crisis responses is due to the downgrading of its importance, hence not requiring coercive retaliatory reactions. Nevertheless, there is a lack of investigation of the cognitive processes related to state crisis responses. This leads to a lack of understanding of the internal processes within a state that allows it to exhibit self-controlled behavior.

This perspective piece attempts to answer the case of self-control exhibited by state actors in international relations. In doing so, it argues that existing studies have not been able to make sense of foreign policy decisions in times of crisis. Thus, bridging social psychology theories to make sense of international relations empirical puzzles is attempted. Specifically, this study references the theory of self-control introduced by Walter Mischel after a series of studies made in the field of social psychology. This study argues that a state’s responses to crisis in the status quo can be comprehended by investigating aspects that contribute to a state’s self-control, primarily from cognitive-affective units. Throughout past decades, Mischel’s studies have decomposed the phenomena of why individuals have thoughts of delayed gratitude for greater rewards in the future. By changing the variable of individuals to state actors, this study will attempt to add to the long list of sociological theories bridged to the study of international relations.

## 2 Control in social psychology: theoretical foundations

In Mischel’s study of self-control in social psychology, the point of inquiry is what goes through the cognitive thoughts of individuals when they decide to pass upon an instant reward for a greater one in the future. The “Marshmellow Test” has become the seminal study in the self-control theory to understand why individuals are willing to delay immediate gratification for a delayed reward. This section will discuss critical elements of Mischel’s study and share several possible convergences of ideas that could be bridged to international relations discourses.

Mischel’s Marshmellow Test took place in Southern Trinidad, with children being the primary subject of analysis. Mischel’s study aimed to decompose why individuals can exercise self-control and whether factors such as beliefs, expectancies, and possible reception of a larger reward would be essential variables in one’s decision to self-control (Mischel, 2012). To conclude, this study assessed the “…delay of immediate gratification for the sake of delayed but more valued rewards paradigm” (Mischel, 2012). The Marshmellow Test thus focused on identifying what cognitive-affective units, in terms of mental processes, were pivotal in a child’s decision to delay immediate gratification.

The study’s initial results pointed to several crucial variables that could explain why some exercised self-control while others did not: goal commitment and expectancy value. In goal commitment, it is argued that the ability of an individual to focus his thoughts on a delayed reward is attributed to how that individual perceives the goal being at stake. Thus, commitment toward a particular goal, whether linked to the delayed reward or not, provides the foundations of an individual’s mental process for self-control. Linked to this variable is the expectancy value. In Mischel’s study, his subjects were given the option of a smaller candy immediately or a larger one later (Mischel, 1973; Mischel and Ayduk, 2004). He concluded that self-control can occur only if an individual values a delayed reward larger than the immediate one. Furthermore, once valued higher, the individual must also have the ability to self-restrain from choosing the immediate reward (Mischel and Staub, 1965; Bandura, 1986).

The conclusions to Mischel’s Marshmellow Test lead to several cognitive-affective units detrimental to the self-control of individuals: Encoding (the construction of self and others), expectations and beliefs of the outcomes, affects (feelings and emotions), goals and values, and competencies and self-regulatory plans (the plan of action to control internally behaviors toward the rewards) (Mischel, 1973). The self-control theory thus provides a nuanced understanding within social psychology discourses on why individuals are able to showcase a commitment to the pursuit of a delayed reward rather than take the immediate reward in front of their eyes.

The following section argues that the self-control theory within sociological discourses can be bridged into international discourses. This perspective article focuses on an empirical puzzle within international relations that may benefit from bridging this theory, which inquiries into the empirical puzzle of why state actors can display self-restraint from aggressive foreign policies in the face of crisis. It argues that Mischel’s cognitive-affective units can be utilized in state actors, slightly modifying how the framework is used for the context of state actors.

## 3 Decomposing self-control in state actors: potential bridging to international relations discourses

Bridging sociological theories into international relations discourses is not a new venture in academia. In the past, seminal studies in constructivism have boosted the relevance of sociological discourses in understanding how the world works. Among the studies is Alexander Wendt’s “Social Theory of International Politics,” which focuses on bridging sociological theories to interpret the international system as a social construct. Thus, when realism tends to reference the state of anarchy as an inevitable occurrence in world affairs, Wendt’s constructivism focuses on how anarchy is “what states make of it” (Wendt, 1999). There has also been a rise in role theory’s usage within international relations studies. Popularized by K. J. Holsti’s seminal study “National Role Conception in the Study of Foreign Policy,” this argues that states adopt certain role conceptions after considering internal and external expectations of states (Holsti, 1970). This conception provides a more nuanced understanding of how states behave in the international system and predicts the adoption of state foreign policies consistent with its role conceptions.

This perspective article argues that a similar bridging of sociological theories can be made to make sense of a state’s responses to crisis. Informed by Mischel’s self-control theory, this study perceives there is great relevance between the cognitive-affective units displayed when individuals exercise self-controlling actions and how a state refrains from taking immediate aggressive foreign policies vis-à-vis crisis. First, it is vital to explain cognitive-affective in terms of state actors. This study argues that the self-control theory’s cognitive-affective units are similar to the internal processes related to state policy-making bodies. This would include how political elites, government stakeholders, and ministries related to a crisis decide to escalate or de-escalate tensions. Similar to individuals, the cognitive-affective units would eventually differentiate among states. Some states would place heavier emphasis on presidents and individual autocratic leaders alone. In contrast, a state with a more democratic setting would have multiple layers of filters to determine crisis responses (thus consisting of many cognitive-affective units).

Encodings, consisting of internal and external constructs of a state, are somewhat already bridged into international relations discourses in the self-identity theory. In this, it is argued that a state constructs its self-identity by considering its internal constituencies’ norms, values, and interests, adding to the expectations of identities perceived by other states. Thus, self-identity in international relations is a relational theory. In the self-control theory of social psychology, encodings are essential in providing the foundational context. This means that to understand future expectations, individuals would need first to construct what is happening based on their cognitive and affective thoughts. Bridged to international relations discourse, this would mean understanding how such a state identified itself in relation to other actors, events, and situations faced. Existing international relations scholarship on self-identity and role conceptions can provide the basis of a state’s encodings vis-à-vis a crisis. A state internally and externally categorized as a “middle power” for example, would perceive itself more under the context of adopting peaceful activism and niche diplomacies, while a great power would perceive itself with the need to adopt decisive and leadership roles in world affairs (Holbraad, 1984; Jordaan, 2003; Patience, 2014; Robertson, 2017; Giang, 2023). Perceptions of power and the state’s position in world affairs are also integral aspects of encodings. Self-control is likely to happen when mutual recognition is present (hard and soft power). Consequently, a state’s encodings would need to be discursively investigated and contextualized.

The second cognitive-affective unit is expectancies and beliefs. In international relations studies, a state actor would have its own beliefs, usually driven by norms that are detrimental to that state. In the process of determining what to do after a crisis, this study argues that states would consider such beliefs first, which would guide the types of expectations held vis-à-vis the crisis. When facing greater power, for example, states tend first to identify what foreign policy norms are relevant to the interaction. This is followed by the emergence of expectancies of possible courses of action that may form. In expectancies, there is an understanding that certain behaviors would lead to certain situations.

Consequently, within state-to-state interactions, states are aware of the consequences of adopting peaceful or coercive responses in the face of crisis. For example, the secondary states of Southeast Asia, due to the past struggles with colonialism, have advocated the norms of non-alignment and self-determination (Emmerson, 1984; Till, 2022; Putra, 2023b). Thus, its actions in international relations are guided by such norms, which leads to expectancies that are relevant to that.

For the variable of goals and values, as with individuals, states tend to adopt specific goals and values that guide the actions taken to achieve such goals. This is usually termed as state “grand strategies” and plan of action for the upcoming years. Unlike the adoption of coercive responses to a crisis, this analysis allows an unpacking of what a state wishes to achieve for its people, which then may explain why specific foreign policies seem to not conform with existing theoretical predictions. For example, with the South China Sea crisis, this cognitive-affective unit allows an investigation into the long-term goals that the claimant states for the contested waters. For the secondary states of Southeast Asia that are claimant states to the disputed waters, their actions could be interpreted as being guided by the goal of maintaining sovereignty in its maritime borders, which is detrimental to the securing of Sea Lanes of Communication and the development of its blue economies (Fravel, 2011; Fravel and Glaser, 2022). Thus, when a crisis occurs, these smaller states tend not to react aggressively, despite the common narrative that China’s intrusions would eventually lead to the loss of sovereignty.

Equally important is the cognitive-affective unit of self-regulatory plans. Understanding that a state has long-term goals and expectancies related to a crisis, this variable inquiries into the possible actions that could be maintained related to the priority for delayed rewards. This cognitive thought considers how states understand that refraining from aggressive foreign policies contains a delayed gratification worthy of being pursued. It also believes that states understand that a response is still needed to manage existing tensions, and the failure to respond to such tensions may lead to the loss of trust within a state’s domestic constituencies. Thus, self-regulatory plans are taken as a means to respond to a crisis in a manner that represents self-controlling in such states to achieve greater future rewards.

If combined, these cognitive-affective units allow for a more nuanced understanding of interpreting a state’s non-coercive response in facing a crisis within international relations discourses. The study of self-control within social psychology has opened up interpretations in understanding why individuals are willing to pursue their long-term goals despite facing immediate temptations. This study argues that insights from self-control theory can contribute to international relations discourses aiming to understand states’ foreign policy decisions in the face of crisis. By considering the units of encodings, expectancies, beliefs, goals, values, and self-regulatory plans of a state, we can understand why state actors also practice the act of self-control in international relations. Indeed, those cognitive-affective units present within an individual decision of immediate or delayed gratification are also present within a state’s thought processes in determining an appropriate course of responses toward a crisis, as states also consider highly the importance of maintaining a commitment to pursue delayed rewards over immediate gratification if the value is seen greater. Eventually, this perspective article builds upon existing academic efforts aiming to bridge sociological discourse to international relations by adopting a sociological understanding of foreign policy decisions in times of crisis.

## Data Availability

The original contributions presented in the study are included in the article/supplementary material, further inquiries can be directed to the corresponding author.
